# The Effect of Short Duration Sperm Exposure on Development of Preeclampsia in Primigravid Women

**Published:** 2012-01-01

**Authors:** Z Sadat, M Abedzadeh Kalahroudi, F Saberi

**Affiliations:** 1 Trauma Research Center, School of Nursing and Midwifery, Kashan University of Medical Sciences, Kashan, Iran; 2 Department of Midwifery, Kashan University of Medical Sciences, Kashan, Iran

**Keywords:** Preeclampsia, Sperm exposure, Cohabitation, Pregnancy

## Abstract

**Background:**

Preeclampsia is one of the most serious complications, and affecting about 3% of pregnancies. The aim of this study was to estimate the impact short duration of exposure to sperm on development of preeclampsia.

**Methods:**

The duration of sperm exposure with the biological father (cohabitation without barrier methods) <3, <6 months were evaluated among 120 primigravid women with preeclampsia and 120 women without preeclampsia in a case-control study.

**Results:**

The short duration of exposure to sperm was more common in women with preeclampsia compared with controls (29.2 versus 14.2 for <3 months, adjOR 2.6 (95% CI=1.32-5.13) and (45 versus 29.2 for <6 months, adjOR 2.4 (95% CI=1.35-4.32). Regardless of the contraceptive method, short duration of cohabitation was more common in preeclamptic group (14.2 versus 5.8 for <3 months, adjOR 3.38 (95% CI=1.28-8.92) and (29.7 versus 13.3 for <6 months, adjOR 2.64(95% CI=1.24-5.79).

**Conclusion:**

It was concluded that short duration of exposure to sperm was more common in women with preeclampsia compared with controls.

## Introduction

Preeclampsia is one of the most serious complications affecting about 3% of pregnancies. It may threaten maternal and perinatal survival.[1][2][3][4] Pre-eclampsia occurs after midgestation, is defined by hypertension (systolic blood pressure =140 mmHg, diastolic blood pressure =90 mmHg) and accompanied by a new onset of proteinuria that is defined by =300 mg per 24 h or =1+ on dipstick testing. Pre-eclampsia is a multisystem disorder, unique to pregnancy.[5] The etiology remains poorly understood, thus allowing little prospect of strategies for prevention. Several risk factors have been associated including previous history of hypertension in pregnancy, family history, body mass index and booking diastolic blood pressure.[6] A number of hypotheses on the etiology and early pathogenesis of preeclampsia are currently popular. One of the hypotheses is the immunogenetic maladaptation and the strongest protective factor for preeclampsia is a prior birth or pregnancy suggesting that immune tolerance may play an etiologic role.[7] A series of epidemiological studies have been published supporting the concept of maternal–fetal immune maladaptation, with development preeclampsia associated with short sperm exposure.[8][9][10] Although others have refuted such relationship.[1][11] A change of paternity , donor insemination and barrier contraception would increases the risk of preeclampsia.[12][13][14] However these results were not confirmed by other researchers.[15][16][17] Thus it remains uncertain whether a short duration of sexual relationship with the biological father prior to conception is associated with preeclampsia. Due to this controversy, the current study was designed to estimate the impact of the short duration of sperm exposure in primigravid on the risk of developing preeclampsia.

## Materials and Methods

An analytic case- control study was performed. The subject population consisted of primigravid women who were delivered at maternity Hospital in Kashan city during 2005-2007. Cases had any degree of preeclampsia, as defined at least blood pressure measurements =14/90 mmHg, and proteinuria of =1+ on dipstick testing. Control was selected by taking the first primigravid patient in the ward who delivered after a patient in the case group with no hypertensive disorder and without any of the exclusion criteria. Patients for the case group were selected by sequential with regard to inclusion and exclusion criteria and sampling in the control group was randomly. We calculated the sample size assuming an a of 0.05, α β of 0.20 (power=80%), and a prevalence of sperm exposure <3 months estimated at 15%. To detect an odds ratio (OR) of 2.5 as significant using a 1:1 ratio between cases and controls, we needed 120 cases and 120 controls. Women booked after 24 weeks’ gestation for cases and term gestation for controls, women to have prenatal care before 20 weeks gestation, singleton pregnancy and the study population was ethnically Iranian. Exclusionary conditions included others preexisting diseases, new paternity, body mass index >30, history of infertility and other pregnancy complications. After obtaining informed consent, all patients were interviewed post partum by the trained midwifes and obtained data on demographics characteristic and duration of sperm exposure (calculated by deducting months of barrier use from total months of the sexual intercourse before, the method of contraception (barrier methods were condoms, and withdrawal). We compared sperm exposure and sexual cohabitation <3, <6 months and >12 months between two groups. Content validity and test-retest was performed to assess validity and reliability of the questionnaires. Data was analyzed by SPSS software (version 16, Chicago, IL, USA). Differences in means were analyzed using the Student's t-test and the Mann–Whitney U-test. The χ(2) and Fisher's methods were applied to qualitative variables. Adjusted odds ratios were computed using a multiple logistic regression model, which included potential confounding factors. P value of less than 0.05 was regarded as significant. The study protocol was approved by the local Research Committee in Kashan University of Medical Sciences.

## Results

Results on the demographics characteristics in women were presented in Table 1. There were no significant differences between cases and controls with regard to these variables but women with preeclamptsia had a higher Body Mass Index (BMI) than controls (p=0.017). Women with preeclampsia were more likely to have a short duration of sperm exposure (adjOR=2.6 (1.32-5.13) for <3 months, adjOR=2.4 (1.35-4.32) for <6 months) compared with those with controls. Frequency of short duration of sexual cohabitation was higher significantly in the case group, adjOR=3.38 (1.28-8.9) for <3months, adjOR=2.6 (1.24-5.79) for <6 months (Table 2). Differences in long duration of sperm exposure, long duration of cohabitation (>12 months) and coital rate were not significant between two groups.

**Table 1:** General characteristics of patients studied in two groups.

**Characteristics**	**Preeclamptic (n=120)**	**Non-preeclamptic (n=120)**	***P *value**
Maternal age	23.2 (3.5)	22.7 (3.1)	0.27
Body mass index	23.8 (2.6)	22.9 (2.7)	0.017
Passive smoking^a^	21 (17.5)	30 (25.0)	0.15
Oral contraception	30 (25)	27 (22.5)	0.68
Barrier contraception	72 (60.8)	69 (57.7)	0.69
Aspirin or calcium usage	20 (16.7)	14 (11.7)	0.26
Sex of fetus			
Male	62(51.7)	65(54.2)	0.7
Female	58(48.3)	55(45.8)	
Level of education			
<8	69(57.5)	61(50.8)	0.3
≥8	51(4.5)	59(49.2)	

^a^ Data are given as mean (SD) or numbers (percent). Non of subjects were smoker, Student t-test and the Mann–Whitney U-test were applied to quantitative variables. The X^2^ and Fisher's methods were applied to qualitative variables.

**Table 2:** Sperm exposure, sexual cohabitation and coital rate before conception in two groups.

**Characteristics**	**Preeclamptic (n=120)**	**Non-preeclamptic (n=120)**	***P* value **	**Adj. OR (95% CI)**
Sperm exposurea				
<3 months	35 (29.2)	17 (14.2)	0.005	2.6 (1.32-5.13)
<6 months	54 (45)	35 (29.2)	0.01	2.4 (1.35-4.32)
>12 months	32 (26.7)	42 (35)	0.20	1.6 (0.89-2.9)
Sexual cohabitation				
<3 months	17 (14.2)	7 (5.8)	0.01	3.38 (1.28-8.9)
<6 months	32 (26.7)	16 (13.3)	0.014	2.6 (1.24-5.79)
>12 months	59 (49.2)	66 (55)	0.30	1.32 (0.77-2.27)
Coital rate/wk before pregnancy^a^	2.8 (1.3)	2.9 (1.5)	0.59	**
Coital rate during pregnancy	1.8 (1.1)	1.9 (1.2)	0.5	

^a^ Data are given as mean (SD) or numbers (percent). Adjusted for age, BMI, aspirin or calcium use, passive smoking, level of education and planned pregnancy. In 2 months before pregnancy. Student t-test and the Mann–Whitney U-test were applied to quantitative variables. The x^2^ and Fisher's methods were applied to qualitative variables.

## Discussion

Results demonstrated that a short duration of sperm exposure and short duration of cohabitation increased the risk of preeclampsia. No relationship was observed between long duration of sperm exposure, coital rate and risk for preeclampsia. These factors failed to recognize the group with a long sexual relationship and a high coital rate but a low exposure to sperm due to prolonged use of barrier contraception. Short duration of sperm exposure is thought to be a better measure for positive predictor of preeclampsia. This may be explained by the achievement of immunological tolerance towards partner's sperm after 6 months of unprotected sexual cohabitation.[18]

Our results for preeclampsia are consistent with several studies.[9][10][18][19] A retrospective case-control study in a group of 68 women of mixed parity with pregnancy-induced hypertensive disorders found that for primiparous women with a shorter duration of sexual cohabitation was not associated with pregnancy-induced hypertensive disorders. However, in this study sample size was small, mixed parity and 20–40% of cases had a history of a previous abortion and some significant fertility limiting factors.[11] In a prospective study of 2211 Pittsburgh population in unadjusted analyses, a prolonged time to conception was associated with pre-eclampsia (OR, 1.9); however, after adjustment, the association was less prominent (OR, 1.6) and, after stratification by contraception method, the link between time to conception and preeclampsia was eliminated. The most important in this data being a first month conception rate of 40–55%, also multiparous women were included in their study; multiparous women were less likely to develop preeclampsia than nullipara.[20] Recently in a prospective study on Nigerian women in their second pregnancies: Results showed there was no significant difference in the incidence of preeclampsia between women who had changed paternity and those without change in paternity. The inter-pregnancy interval and the mean duration of sexual cohabitation were similar between women who had changed paternity that developed preeclampsia and those that did not developed preeclampsia. In this study, multiparous women and new partners were included; there was a complex interaction between changing of partners and the inter-pregnancy interval[17] whereas our study comprised only primigravid without new paternity. In a case–control study,[21] there was no significant difference between women with pre-eclampsia and their controls in respect to the duration of cohabitation which was less than 12 months prior to conception, though 24.5% of late onset pre-eclamptics had a period of cohabitation less than 12 months compared with only 8.6% of the controls. Also in their study, 12 months cut-off was considered as a short periods of sperm exposure to seminal fluids before conception could result in down-regulation of the mother's immune response to foreign antigens, thereby reducing the risk for development of preeclampsia. The protective effect of a more lengthy sperm exposure could be explained by so-called maternal mucosal tolerance to paternal antigens.[22] The cellular cytokine responses in human vaginal and cervical cells have recently been elucidated. Additionally, one of the explanations for contradictory findings might be that the type of preeclampsia involves 30 and more years old, mildly obese pregnant women having near-term preeclampsia is primarily related to pre-existing maternal constitutional factors. In contrast, the epidemiology of preeclampsia in young women (age 15–25 years), so commonly appears more in line with the immune maladaptation hypothesis. Limited semen exposure may be the most likely explanation for the high incidence of preeclampsia in teenagers.[23]

This study suggests a relationship between short duration of sperm exposure also short duration of cohabitation, and risk for preeclampsia. Strengths in this study were high quality data which were obtained from the participants by trained questionnaires after delivery. Sample size estimation was based on power calculation and patients were ethnically Iranian and we used multivariate analysis for controlling recognized risk factors for preeclampsia. However, limitations in our study were sexual practices that might not be accurately reported especially in women with long duration of cohabitation before pregnancy. We also considered it difficult to collect reliable data on oral sex therefore, we cannot account for exposure to seminal fluid through this route. In conclusion, short period of exposure to sperm (<6 months) seem to be at elevated risk for development of preeclampsia. However, limitations of this study could be as a plan for primigravid women that can be recommended to prolong sexual cohabitation at least 6 months before pregnancy without barrier contraception in order to decrease the risk of preeclampsia.
